# Liposomal co-delivered oleanolic acid attenuates doxorubicin-induced multi-organ toxicity in hepatocellular carcinoma

**DOI:** 10.18632/oncotarget.17559

**Published:** 2017-05-02

**Authors:** Muhammad Sarfraz, Attia Afzal, Shahid Masood Raza, Sajid Bashir, Asadullah Madni, Muhammad Waseem Khan, Xiang Ma, Guangya Xiang

**Affiliations:** ^1^ School of Pharmacy, Tongji Medical College, Huazhong University of Science and Technology, Wuhan, 430030, Hubei, China; ^2^ Department of Pharmacy, University of Sargodha, Sargodha, 40100, Punjab, Pakistan; ^3^ Institute of Pharmacy, Lahore College for Women University, Lahore, 54610, Punjab, Pakistan; ^4^ Faculty of Pharmacy and Alternative Medicine, The Islamia University of Bahawalpur, Bahawalpur, 63100, Punjab, Pakistan

**Keywords:** oleanolic acid, doxorubicin, cardiotoxicity, 20-HETE, HCC

## Abstract

Doxorubicin in combination with other cytotoxic drugs has clinical advantages. However, doxorubicin-induced cardiotoxicity negatively impacts clinical utility and outcomes. Cardiotoxicity can result from increased oxidative stress or from a local cytochrome P450 mediated increase in 20-hydroxy-5, 8, 11, 14-eicosatetraenoic acid (20-HETE). Oleanolic acid (OA) is a natural pentacyclic triterpenoid with free radical scavenging, cardioprotective, and P450-mediated cyclooxygenase-upregulating properties. We investigated co-delivery of liposomal OA and doxorubicin in a HepG2 model of hepatocellular carcinoma (HCC). OA attenuated the cardiotoxicity induced by doxorubicin without compromising its anticancer activity. Apoptosis assays revealed that co-delivery of DOX and OA produced a synergistic anticancer effect. However, the drugs had antagonistic effects on cardiomyocytes. Female BALB/c nude mice treated with OA- and DOX-loaded liposomes (ODLs) exhibited reduced tumor growth, stable body weight, and stable organ indices. Reduced 20-HETE production suggested ODLs had limited cardiotoxicity. No changes in biochemical or histopathological markers were observed in mice treated with ODLs. Tailored co-delivery of OA and DOX may thus be an effective therapeutic strategy for treating HCC.

## INTRODUCTION

Hepatocellular carcinoma (HCC) is the sixth most common cancer worldwide [1]. Single-agent chemotherapy is not sufficient to prevent reoccurrence because of tumor heterogeneity and the complexity of cell signaling pathways. Targeted and combination therapies are frequently used to treat HCC and many regimens have been investigated in clinical trials [2]. Adjuvant therapies may improve patient outcomes [3, 4]. Chemotherapeutics in combination with natural compounds is an attractive approach for HCC treatment [5].

The anthracycline anticancer drug doxorubicin (DOX) is effective for various malignancies but it can cause cardiotoxicity. The mechanisms underlying DOX-induced cardiotoxicity are distinct from those responsible for the therapeutic effects [6]. Cardiotoxicity is caused by increased oxidative stress and free radical formation (reactive oxygen species, ROS) [7–10]. DOX-induced 20-hydroxy-5, 8, 11, 14-eicosatetraenoic acid (20-HETE) production [11] promotes cardiomyocyte apoptosis through the intrinsic (mitochondrial) pathway, which can damage the heart [12].

Oleanolic acid (OA), a natural pentacyclic triterpenoid, has cardioprotective effects when administered after an ischemic insult [13]. The OA-mediated cardioprotective effects might reflect a direct scavenging role (i.e. by decreasing superoxide and hydrogen peroxide levels) and reduced lipid peroxidation [14]. COX-2 activation is also involved in OA-induced HepG2 cell cycle arrest and apoptosis. Activation of COX-2 through increased PGE_2_ and PGI_2_ production (vasodilator prostanoids) [15, 16] likely counteracts the vasoconstrictor response of 20-HETE. Thus, OA (a lipophilic compound) could protect myocardial cell membranes from DOX-induced oxidative stress through direct (ROS) or indirect (20-HETE) pathways, without compromising the anticancer activity.

Combined OA and DOX treatment is limited by the pharmacokinetics, which can lead to a non-uniform distribution and an insufficient dose delivered to the tumor [17]. OA is a Biopharmaceutics Classification System Class IV drug with low aqueous solubility and low permeability across the intestinal mucosa, which restricts its absorption and bioavailability [18, 19]. Liposomes have high loading capability for lipophilic (e.g. OA) and hydrophilic drugs (e.g. DOX) in their outer lipid and inner aqueous compartments, respectively. Liposomes have several advantages including enhanced absorption capability, biocompatibility, biodegradability, low toxicity, and the ability to improve the physicochemical properties of labile or insoluble drugs [20, 21]. We investigated the anticancer effects of OA and DOX, and assessed the effectiveness of tailored co-delivery of liposomal DOX and OA in a HepG2 mouse model of HCC. Additionally, we analyzed whether OA could attenuate DOX-induced cardiotoxicity.

## RESULTS

### Cytotoxicity and survival studies

**Figure 1:**
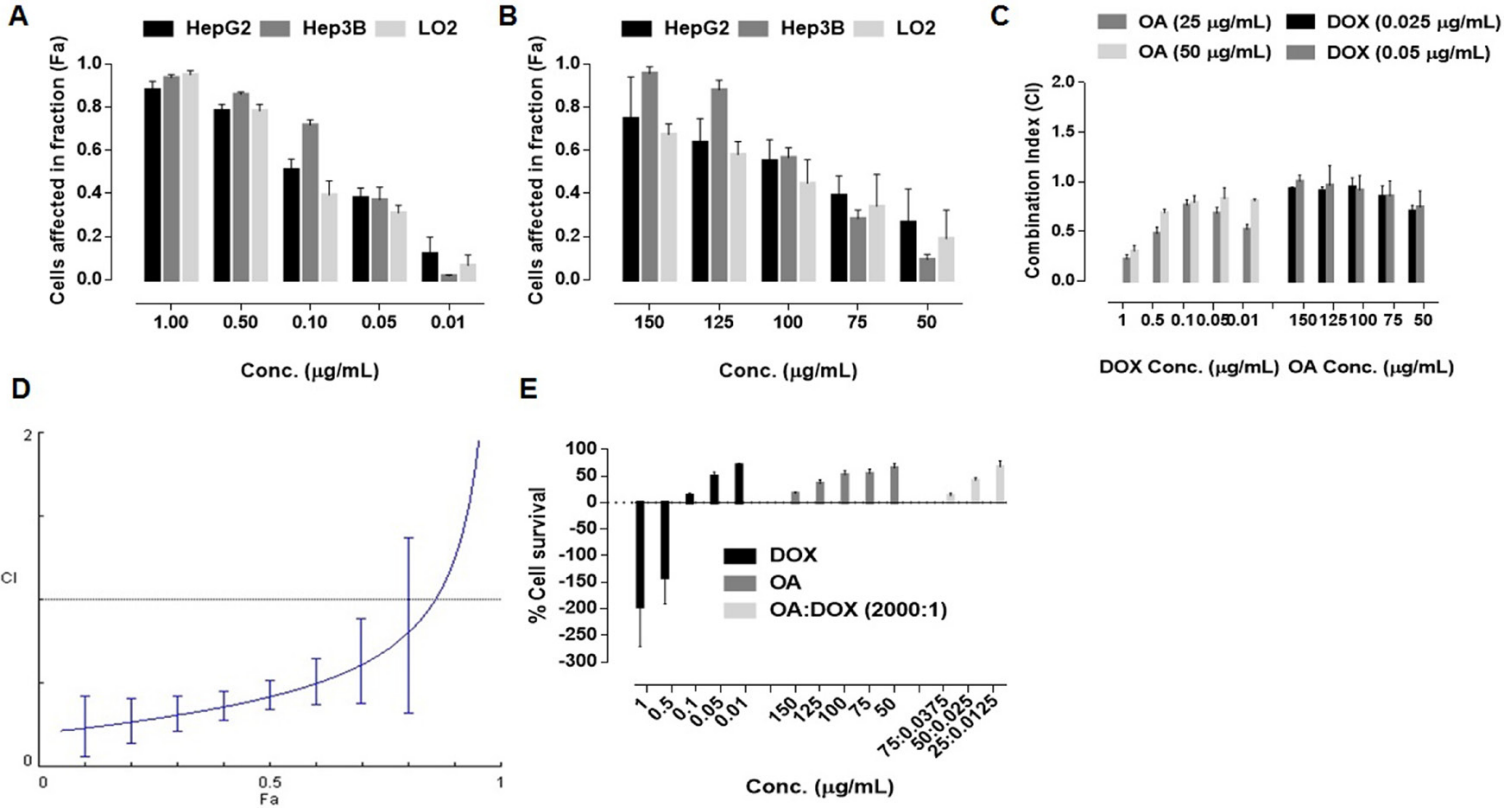
Dose dependent cell killing effects after 72 h exposure of drug treatment (*n* = 5) (**A**) free DOX (**B**) free OA (**C**) Combination Indices at non fixed ratio (**D**) Combination indices at fixed ratio (2000:1, OA:DOX, w/w) (**E)** Cell survival after 72 h of 72 h drug exposure.

We evaluated cell viability after exposure to DOX and OA at various concentrations. All cell lines exhibited dose-dependent cell death in response to DOX exposure (Figure 1A). The IC_50_ values were 0.098 ± 0.013 μg/mL, 0.12 ± 0.01 μg/mL, and 0.174 ± 0.021 μg/mL for HepG2, Hep3B, and L02 cells, respectively). OA was less active than DOX (the IC_50_ values were 78.03 ± 5.03 μg/mL, 64.49 ± 4.71 μg/mL, and 110.43 ± 10.62 μg/mL for HepG2, Hep3B, and L02 cells, respectively) (Figure 1B). Fixed and non-fixed drug combination studies were performed in HepG2 cells. The cells were treated with non-fixed ratios of OA:DOX and the combination index (CI) was calculated. Cell viability was ratio-dependent. The CI values were all < 1.0, indicating synergism between the two drugs (Figure 1C). A fixed ratio of 2000:1 OA:DOX (w/w) had the greatest effect (Figure 1D). The survival rate was approximately 50% after treatment with a single dose of OA (75 μg/mL) or DOX (0.0375 μg/mL) (Figure 1E). In contrast, the survival rate after combined treatment with DOX and OA at a ratio of 2000:1 OA:DOX was only 5%. Hence, combination therapy was 10-fold more cytotoxic than treatment with either drug alone.

### Liposome preparation

We prepared nine liposomal formulations of OA and DOX at a fixed ratio of 5:1 OA:DOX (w/w). The formulations were developed under the influence of four factors at three levels and resulted in various vesicular packages. The L9 (3^4^) orthogonal design is shown in Tables 1 and 2. The entrapment efficiency (EE) was 60–92% for OA and 47–95% for DOX. The combined average EE was 53–89% (Table 2). The particle size (PS) was 85–200 nm in all formulations. Therefore, EE was the only factor considered when selecting the experimental design. A2B3C3D2 was considered the best choice for developing the final formulations. The net influence of the experimental factors according to EE, was A > B > C > D (Table 2). Given that the safety margin of ethanol could impact the cell culture results, A2B3C3D2 was modified to A2B1C1D2, which was optimized to ensure higher loading and performance. The EE was > 90% in the final liposome formulations using the A2B1C1D2 design. A PS of 100–200 nm was achieved without further extrusion. The polydispersity index (PDI) was < 0.3. The negative charge (zeta potential, ZP) was calculated for each batch (Table 3).

**Table 1:** Level of experimental factors

Levels	HSPC:CHOL:DSPE. PEG2000 (A)	Drugs:Lipids (B)	Ethanol (%) (C)	Temperature (°C) (D)
1	68:27:5	1:8	Zero	43
2	64:31:5	1:12	1	48
3	57:38:5	1:17	2.5	53

**Table 2:** Orthogonal experimental drug design (OEDD)

	A	B	C	D	PS	ZP	PDI	% EE (OA)	% EE (DOX)	% EE (Comb.)	Range (PS-EE)	Batch ID
Cumulative impact of the study factors on physicochemical attributes at the stated level	1	1	1	1	119.73 ± 7.27	−18.26 ± 5.22	0.17 ± 0.08	59.73 ± 4.21	46.77 ± 3.15	53.25 ± 3.68	66.49	A1B1C1D1-F1
	2	1	2	2	86.27 ± 22.02	−23.49 ± 8.72	0.19 ± 0.02	70.61 ± 3.56	62.60 ± 3	66.60 ± 3.28	19.66	A1B2C2D2-F2
	3	1	3	3	103.93 ± 7.15	−18.52 ± 10.38	0.24 ± 0.03	69.41 ± 2.84	89.78 ± 1.03	79.60 ± 1.93	24.34	A1B3C3D3-F3
	4	2	1	3	88.17 ± 10.19	−16.37 ± 3.23	0.26 ± 0.04	79.63 ± 4.61	79.35 ± 3.46	79.49 ± 4.04	8.68	A2B1C2D3-F4
	5	2	2	1	121.52 ± 13.93	−25.40 ± 3.01	0.28 ± 0.01	78.50 ± 8.63	85.42 ± 3.95	81.96 ± 6.29	39.56	A2B2C3D1-F5
	6	2	3	2	117.27 ± 6.88	−19.02 ± 5.89	0.33 ± 0.03	91.84 ± 1.66	85.37 ± 18.24	88.60 ± 9.95	28.66	A2B3C1D2-F6
	7	3	1	2	147.53 ± 10.72	−20.44 ± 12.76	0.32 ± 0.02	81.53 ± 3.68	71.13 ± 6.57	76.33 ± 5.12	71.20	A3B1C3D2-F7
	8	3	2	3	194.70 ± 10.29	−17.09 ± 3.5	0.34 ± 0.04	77.67 ± 3.59	50.24 ± 8.64	63.95 ± 6.12	130.75	A3B2C1D3-F8
	9	3	3	1	176.37 5.82	−13.86 ± 1.91	0.31 ± 0.03	65.30 ± 4.93	94.77 ± 11.15	80.03 ± 8.04	96.33	A3B3C2D1-F9
**K1**	199.45	209.07	205.8	215.24	K1 is the sum of %EE (Comb.) at level 1							
**K2**	250.05	212.51	226.12	231.53	K2 is the sum of %EE (Comb.) at level 2							
**K3**	220.31	248.23	237.89	223.04	K3 is the sum of %EE (Comb.) at level 3							
**k1**	66.48	69.69	68.6	71.75	k1 is the average of K1							
**k2**	83.35	70.84	75.37	77.18	k2 is the average of K2							
**k3**	73.43	82.74	79.3	74.35	k3 is the average of K3							
**R**	16.87	13.05	10.7	5.43	R is the range difference at three levels (k1,k2,k3)							
	**A2**	**B3**	**C3**	**D2**	A possible best drug design A2B3C3D2							

**Table 3:** Physicochemical attributes of developed liposomal formulations

	ZP	PDI	EEOA (%)	EEDOX (%)	EEComb. (%)
EL	−12.14 ± 5.35	0.12 ± 0.03	−	−	−
OAL	−15.38 ± 1.53	0.18 ± 0.04	98 ± 1.4	−	−
DXL	−19.18 ± 2.01	0.14 ± 0.03	−	91.66 ± 3.23	−
ODL	−17.87 ± 5.72	0.20 ± 0.06	95.13 ± 1.92	92.64 ± 2.26	93.89 ± 2.09

### Liposome characterization

**Figure 2:**
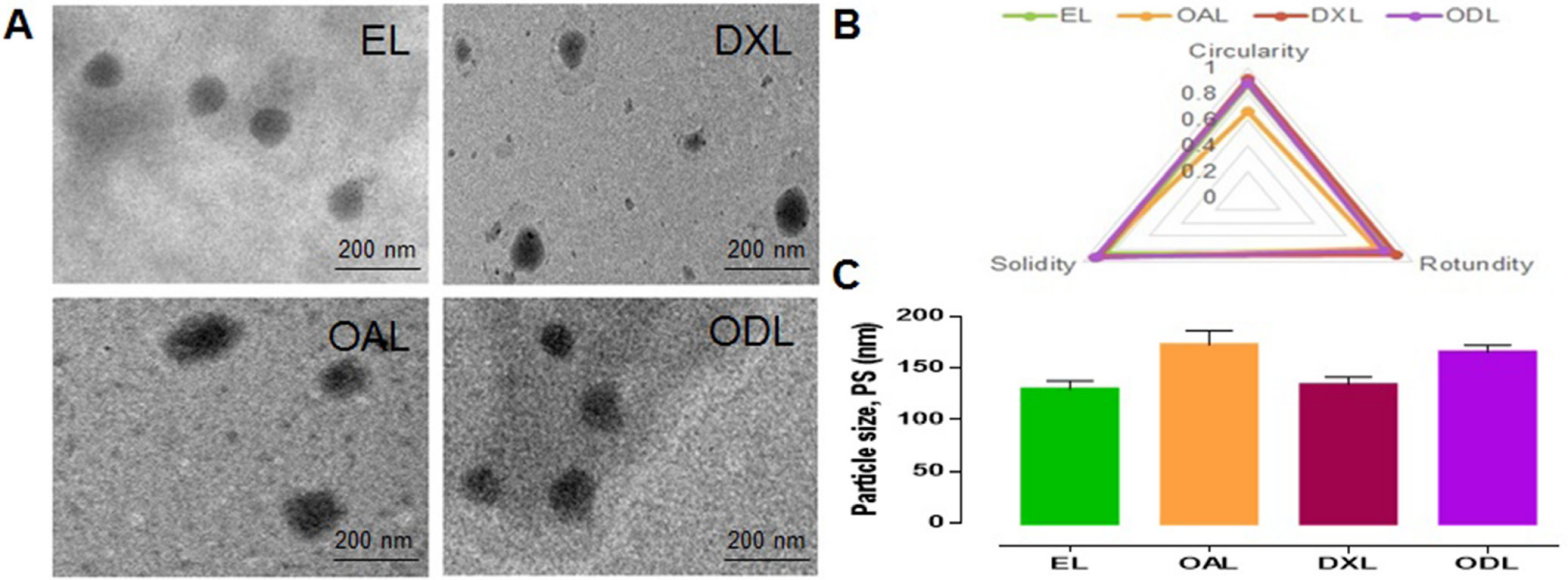
Morphology and physical attributes of developed liposomes (**A**) TEM images at 75 KV, 30000× (**B** and **C**) Physical attributes calculated by Image J (https://imagej.nih.gov/ij/).

Liposomes were imaged by transmission electron microscopy (TEM) and analyzed using Image J (https://imagej.nih.gov/ij/) (Figure 2A). The circularity, rotundity, and solidity were > 0.9 for all formulations with the exception of OA-loaded liposomes (OAL) (0.67 ± 0.038, 0.77 ± 0.045, and 0.92 ± 0.01, respectively) (Figure 2B). Thus, the morphology of OAL was poor. The PS was < 200 nm (Figure 2C).

### Liposome stability

**Figure 3:**
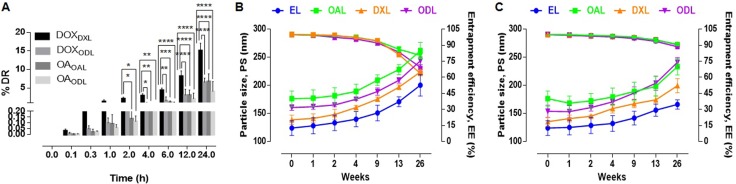
Short term stability studies in various conditions (*n* = 3) (**A**) % Drug leakage in serum (20% FBS in 10 mM HEPES, pH 7.4 at 37°C) (**B**) Impact on PS and % EE after 6 months storage at RT (**C**) Impact on PS and % EE after 6 months storage at 4°C.

We analyzed the stability of DOX-loaded liposomes (DXL), OA- and DOX-loaded liposomes (ODL), and OAL to determine whether they retained their physiological structures. DOX leakage from DXL and ODL was observed within 24 h (15.14 ± 1.95% and 6.55 ± 1.14%, respectively). OA leakage from OAL and ODL did not differ (6.79 ± 2.04% and 4.05 ± 2.7%, respectively, p > 0.05) (Figure 3A).

In order to account for vesicle aggregation and drug leakage, we assessed the stability of liposomes stored at room temperature or 4°C for 6 months (26 weeks) (Figure 3B and 3C). The PS and EE were calculated at various times. An approximately 61% increase in the PS of EL and DXL, and an approximately 48% increase in the PS of OAL, were observed after 26 weeks of storage at RT. The EE of OAL, DXL, and ODL decreased by approximately 19%, 36%, and 30%, respectively, during the study period. After storage at 4°C for 6 months, the percent increase in PS was approximately 40%, 32%, and 47% for OAL, DXL, and ODL, respectively. The percent decrease in EE was approximately 9%, 9%, and 11% for OAL, DXL, and ODL, respectively.

### Drug release

Drug release from liposomes was investigated in media and two different buffers: (1) Dulbecco's Modified Eagle's medium (DMEM) supplemented with 10% fetal bovine serum (FBS), (2) 10 mM HEPES pH 7.4, and (3) 10 mM HEPES pH 4.5. Sustained release of the drugs from liposomes was observed in all solutions. The release of encapsulated DOX was higher in DMEM than in 10 mM HEPES buffer. The percent DOX release from DXL (DOX_DXL_) was higher at pH 4.5 than at pH 7.4. The DOX_DXL_ after 12 h in DMEM, pH 4.5, or pH 7.4 buffer was 56.49 ± 4.49%, 35.37 ± 5.46%, and 2.66 ± 2.55%, respectively. No differences were observed in the percent DOX release from ODL (DOX_ODL_) compared to DOX_DXL_. The DOX_ODL_ after 12 h in DMEM, pH 4.5, and pH 7.4 buffer was 57.4 ± 10%, 39.18 ± 1.12%, and 4.21 ± 1.78%, respectively. The largest DOX_DXL/ODL_ was observed in DMEM, followed by pH 4.5 and pH 7.4 buffer.

**Figure 4:**
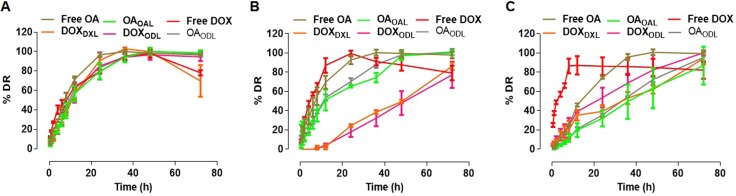
% Drug release in various medium (*n* = 3) (**A**) Complete DMEM with 10% FBS) (**B**) 10 mM HEPES, pH 7.4; (**C**) 10 mM HEPES, pH 4.5.

OA release from liposomes was also highest in DMEM. The percent OA release from OAL (OA_OAL_) in DMEM, pH 4.5, and pH 7.4 buffer after 12 h was 56.68 ± 7.85%, 19.95 ± 7.37%, and 51.31 ± 10.83%, respectively. The percent OA release from ODL (OA_ODL_) after 12 h was 61.64 ± 11.24%, 21.06 ± 1.62%, and 53.62 ± 3.18% in DMEM, pH 4.5, and pH 7.4 buffer, respectively. The highest OA release from liposomes was observed in DMEM followed by pH 7.4 and pH 4.5 buffer. The DOX_DXL_ and DOX_ODL_ in DMEM, pH 4.5, and pH 7.4 buffer after 24 h was approximately 91%, 39%, and 25%, and approximately 85%, 54%, and 18%, respectively. The OA_OAL_ and OA_ODL_ in DMEM, pH 4.5, and pH 7.4 buffer after 24 h was approximately 79%, 32%, and 65%, and approximately 83%, 36%, and 70%, respectively (Figure 4A–4C).

### *In vitro* anticancer activity of liposomes

**Figure 5:**
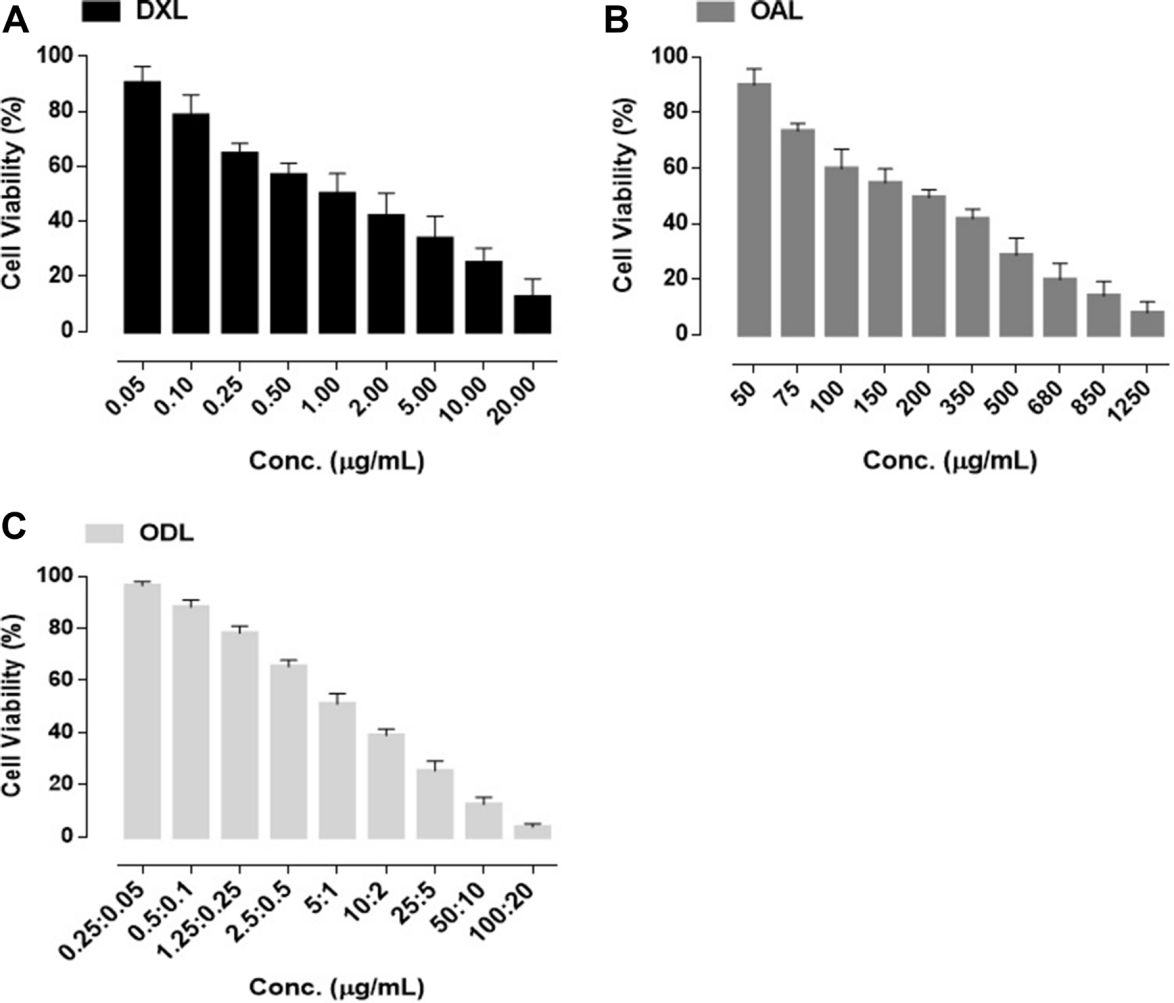
% Cell viability after 72 h exposure of liposomal formulations (**A**) DXL (**B**) OAL (**C**) ODL.

We evaluated cell viability at different doses: DXL (0.05–20 μg/mL), OAL (50–1,250 μg/mL), and ODL (0.25 to 100:0.05 to 20 μg/mL, OA:DOX, respectively). We observed a dose-dependent cytotoxic effect with liposome treatment (Figure 5). Combined delivery resulted in improved anticancer activity. The IC_50_ values were 1.91 ± 0.14 μg/mL, 189.46 ± 20.82 μg/mL, and 1.64 ± 0.089 μg/mL for DXL, OAL, and ODL, respectively.

### Apoptosis assays

**Figure 6:**
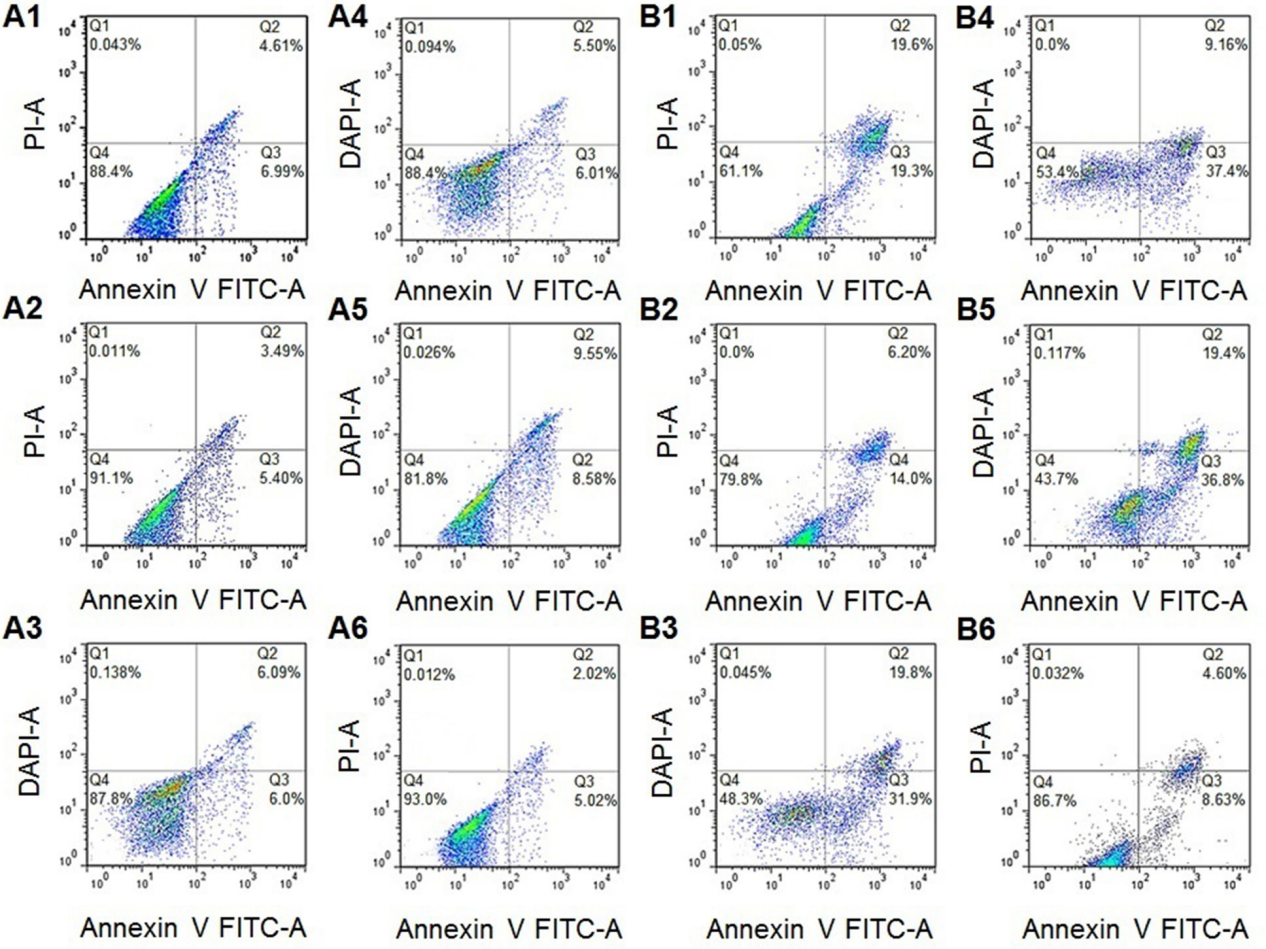
Apoptosis rates of HepG2 cells after (**A**) 24 h (**B**) 48 h; (1. Free OA; 2. OAL; 3. Free DOX; 4. DXL; 5. ODL; 6. Control).

The percentage of viable cells decreased after drug treatment compared to controls (Figure 6). Increased apoptosis was observed after 24 h in the ODL (18.2%) compared to the other groups (Figure 1, 2, 3, 4, 5, 66). A three-fold increase in the rate of apoptosis was observed as the ODL exposure time was increased from 24 h to 48 h (Figure 6B5). The rates of apoptosis were lowest in the OAL group (8.9% and 20.2% after 24 h and 48 h of exposure, respectively) (Figures 6A2 and 9B2). Free DOX was highly cytotoxic. The rates of apoptosis were 12.2% and 51.7% after 4 h and 12 h, respectively (Figure 6A3 and 6B3).

### *In vivo* antitumor assays and end-point bio-distribution studies

The antitumor effects of the liposomes were investigated in HepG2 tumor-bearing female BALB/c nude mice following biphasic drug administration. In the first phase, mice were intravenously (i.v.) injected with liposomes via the tail vein once per day for 4 consecutive days. In the second phase, three doses were administered intraperitoneally (i.p.) every fourth day.

**Figure 7:**
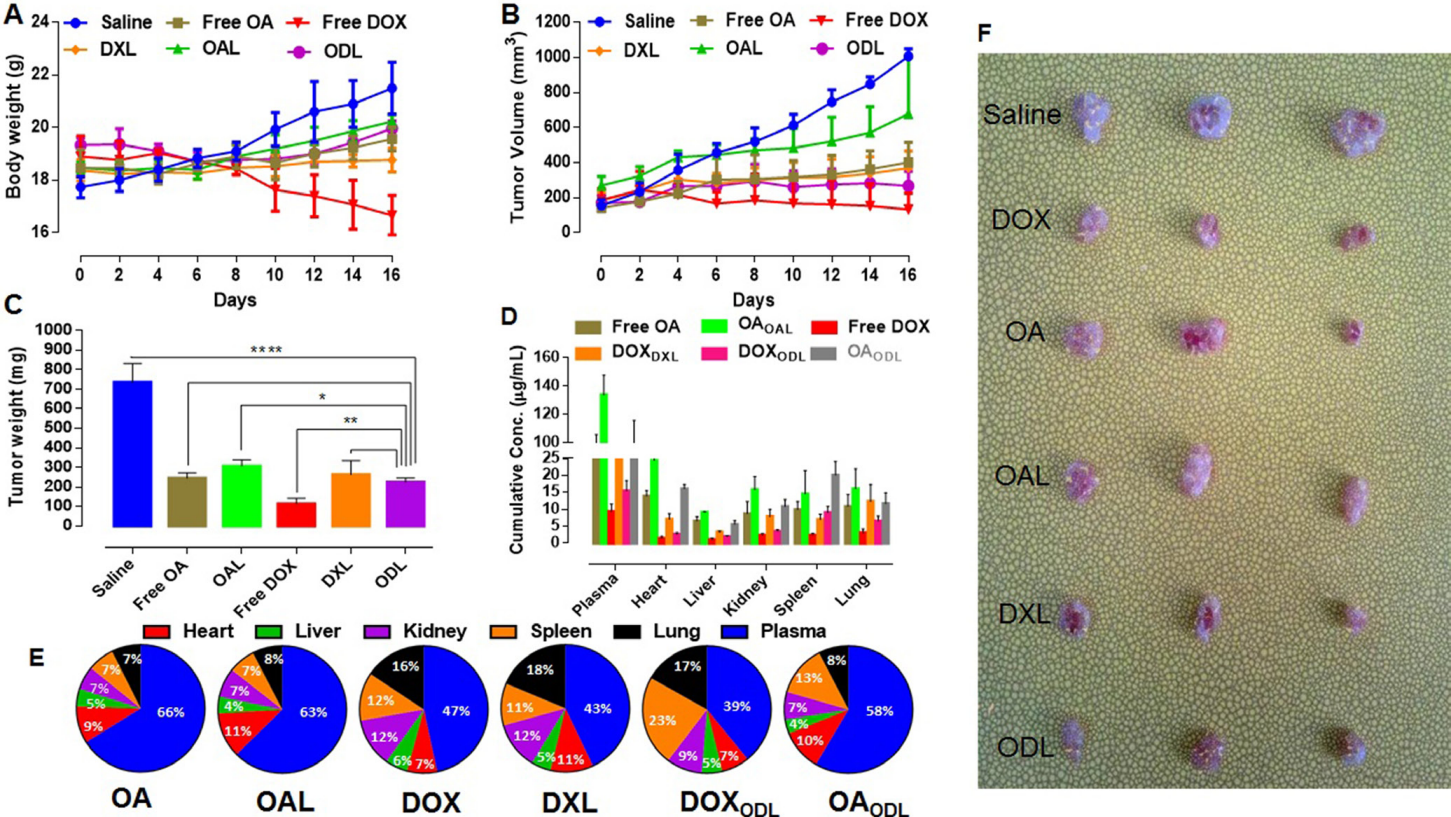
*In vivo* anti-tumor efficacy of developed formulations after consecutive four i.v. injections followed by three i.p. doses with interval of four days (*n* = 3). (OA and OAL = 35 mg/kg, DOX and DXL = 7 mg/kg and ODL = 20:4 mg/kg (OA:DOX; 5:1, w/w) on HepG2 tumor bearing female BALB/c nude mice (**A**) Body weight gain/loss (**B**) Tumor growth curve (**C**) Mass changes in tumor (**D**) Drug distribution in various compartments after 24 h of last injection (**E**) % Drug distribution in bio-compartments (plasma, heart, liver, kidney, spleen and lung) after 24 h of last injection (**F**) Combined photograph of all tumors representative of various treatments.

A gradual loss in body weight was observed in free DOX-treated mice. However, no differences were observed in the other treatment groups. A loss in body weight was also observed in mice treated with ODL during the i.v. phase, but the weight returned to baseline during the i.p. phase (Figure 7A). Tumor volume versus time profiles for the various treatment groups are shown in Figure 7B. Tumor growth was reduced in free OA-treated mice during the i.v. phase. However, gradual tumor growth was observed during the i.p phase. OAL was the least effective of the treatment regimens. However, it reduced tumor growth compared to saline-treated control mice. Free DOX was the most effective against tumor growth. Inhibition of tumor growth was observed in mice treated with DXL during the i.p phase. A small increase in tumor volume was observed in mice treated with ODL during the first half of the study period, but no further growth was observed during the second half of the study.

Tumor weight measurements indicated that the ODL formulation better controlled tumor growth than DXL or OAL. A reduction in tumor mass was observed in mice treated with ODL compared to control mice and mice treated with OAL (p > 0.0001 and p > 0.05, respectively). Although, there was no difference in tumor mass between mice treated with DXL and ODL, DXL was more toxic than ODL. The greatest effects on tumor weight were observed with free DOX, followed by ODL, free OA, DXL, and OAL (Figure 7C). We calculated organ indices to assess toxicity. Representative data are shown in Table 4. DOX exhibited dose-dependent toxicity in the liver, kidney, and heart as indicated by changes in the organ indices relative to the ODL group.

**Table 4:** Changes in organ indices of female BALB/c nude mice after various drug treatments (mean ± SD) (*n* = 3)

Treatment	Liver index (mg/g)	Kidney index (mg/g)	Heart index (mg/g)
Saline	73.01 ± 3.39**	7.82 ± 0.26*	6.07 ± 0.6
OA	66.67 ± 5.87	7.02 ± 0.59	5.99 ± 0.53
OAL	71.99 ± 6.87*	7.56 ± 0.36	6.15 ± 0.62
DOX	52.98 ± 9.57**/^####^	6.42 ± 0.42*/^##^	4.42 ± 0.31**/^##^
DXL	60.26 ± 5.14^###^	6.77 ± 0.65/^#^	5.6 ± 0.63
ODL	63.18 ± 3.87	7.17 ± 0.68	5.98 ± 0.44

*p < 0.05, **p < 0.01, ***p < 0.001, ****p < 0.0001 compared with ODL, ^#^*p <* 0.05, ^##^*p <* 0.01, ^###^*p <* 0.001, ^####^*p <* 0.0001 compared with saline.

We estimated the net drug concentration of the drugs in the blood and organs 24 h after the last injection. Free drugs or liposome-encapsulated drugs were detected in plasma at OA, OAL, DOX, DXL, DOX_ODL_, and OA_ODL_ concentrations of 98.078 ± 7.81 μg/mL, 133.96 ± 13.86 μg/mL, 9.39 ± 2.25 μg/mL, 28.42 ± 7.72 μg/mL, 15.50 ± 2.98 μg/mL, and 90.92 ± 24.94 μg/mL, respectively (Figure 7D). Increased free and encapsulated OA (OA_OAL_,OA_ODL_) were observed in the heart (9%, 11%, and 10%, respectively) and spleen (7%, 7%, and 13%, respectively) compared to the other tissues. Increased DOX_DXL_ was observed in the lung (18%) relative to the other compartments, which showed an approximately equal distribution. Increased DOX_ODL_ was observed in the spleen (23%) and lung (17%). The drug distribution in various bio-compartments is shown in Figure 7E.

### Toxicity

### *Ex vivo* toxicity

**Figure 8:**
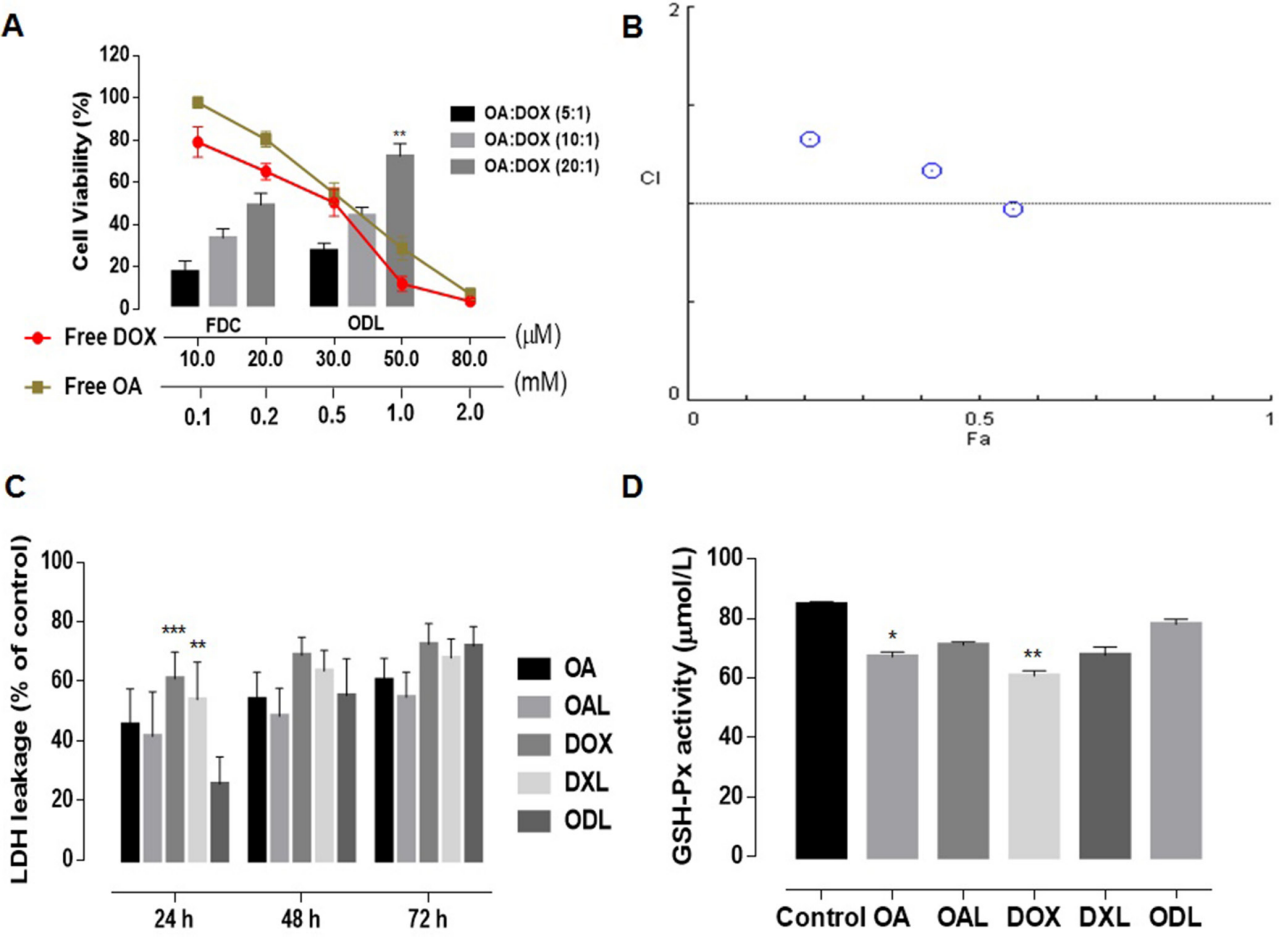
*Ex vivo* toxicity evaluation (*n* = 5) (**A**) cell viability of H9C2 cells (cardiomyocytes) after 48 h of drug treatments; (**B**) Combination indices at non-fixed ratio (**C**) Time-dependent LDH leakage from HepG2 cells against various drug treatments; (**D**) Extracellular GSH-Px activity in HepG2 cell supernatant after 24 h drug exposure. The results were compared with ODL formulation and represented as: *P* < 0.05 (*), *P* < 0.01 (**), *P* < 0.001 (***), *P* < 0.0001 (****).

Because DOX induces cardiotoxicity, we investigated the effects of combined DOX and OA treatment on H9C2 cardiomyocytes. We compared the cytotoxicity of free DOX with OA, free drugs in combination (FDC), and ODL. MTT assays revealed that free DOX had dose-dependent toxicity. It was 25 times more cytotoxic than OA (IC_50_ DOX = 21.2 ± 2.47 μM and OA = 540 ± 25 μM) (Figure 8A). Although a reduction in cell viability was observed with FDC compared to treatment with either drug alone, the CIs were all > 1, indicating the drugs had antagonistic effects in H9C2 cells (Figure 8B). Higher cell viability (26.34 ± 3.64%, 43.4 ± 3.99%, and 72.09 ± 5.97%) was observed with the ODL formulations compared to the FDC (16.11 ± 5.19%, 32.44 ± 4.47%, and 48.35 ± 5.75%) at a ratio of 5:1, 10:1, or 20:1 OA:DOX (w/w), respectively (Figure 8A).

Lactate dehydrogenase (LDH) leakage was analyzed to investigate the membrane integrity of HepG2 cells after exposure to the drugs at the IC_50_ concentrations. Normal saline was used as a control. LDH leakage was time-dependent. Higher LDH leakage was observed after DOX treatment (free and liposomal) (Figure 8C). The percent release of LDH after treatment with OA was 45.46 ± 12.02% of the control after 24 h and 60.43 ± 7.32% after 72 h. Although the LDH leakage after exposure to ODL for 24 h was only 25.55 ± 9.1% of the control, which was less than in the other treatment groups, it increased to 71.84 ± 6.51% after 72 h. ODL was the most toxic liposomal formulation in HepG2 cells after 72 h exposure, followed by DXL, and OAL (Figure 8C).

We analyzed glutathione peroxidase (GSH-Px) activity to assess protection against ROS. A reduction in the GSH-Px level was observed in HepG2 cell supernatants after 24 h of exposure to DOX (free and liposomal). However, GSH-Px activity was limited in HepG2 cells treated with ODL (Figure 8D).

### *In vivo* toxicity

**Figure 9:**
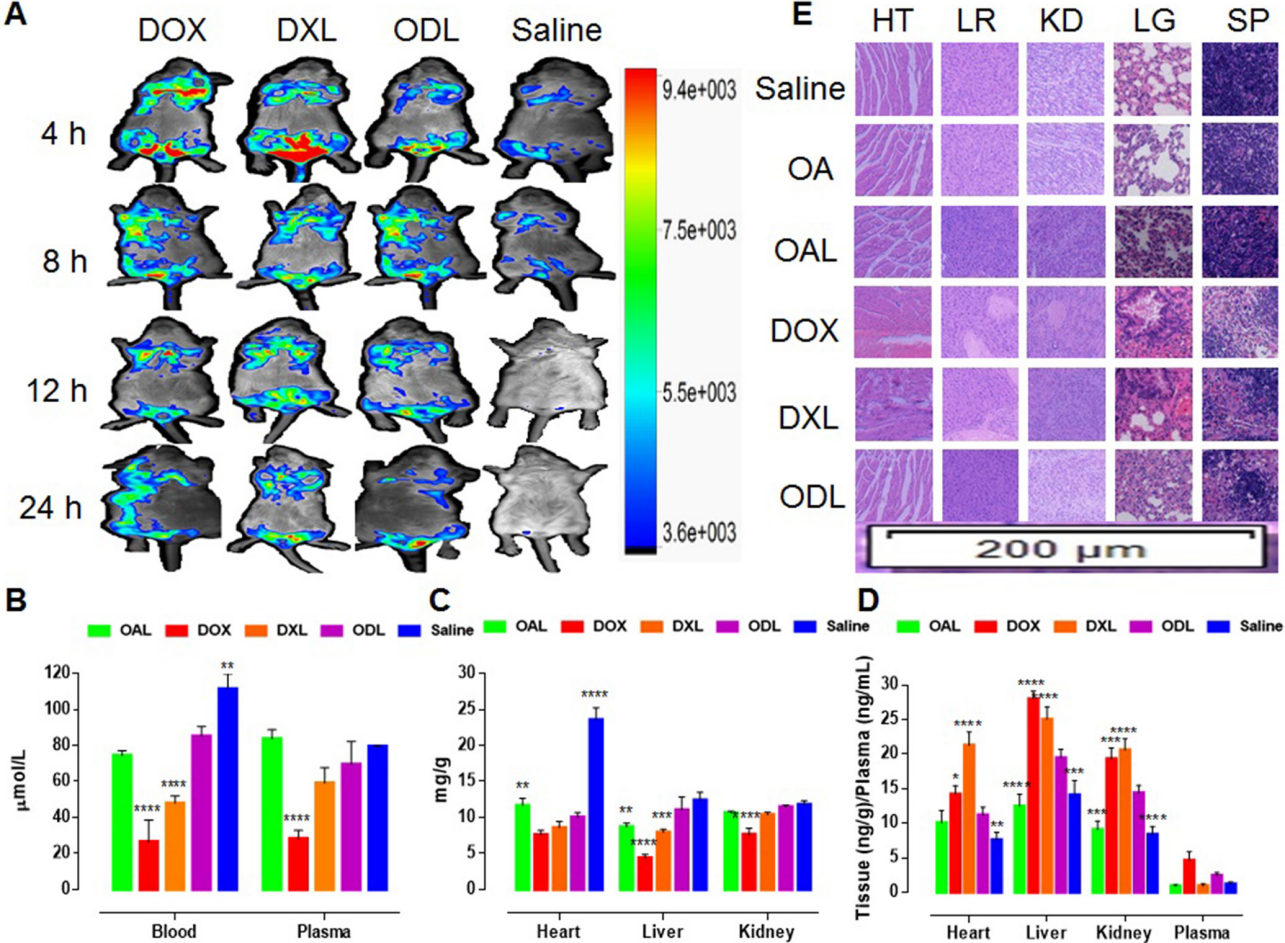
Fate of DOX and *in vivo* toxicity evaluation after i.v. administration of liposomal formulations equal to toxic dose (DOX = 15 mg/kg), via tail vein of Kunming mice (*n* = 3) (**A**) *In vivo* DOX signaling in anesthetized Kunming mice (**B**) GSH-Px activity in whole blood and plasma after 24 h (**C**) GSH-Px activity in heart, liver and kidney; (**D**) Production of arachidonic acid metabolite (20-HETE) after 24 h (**E**) Histopathological evaluation of body organs (HT: heart; LR: liver; KD: kidney; SP: spleen; LG: lung) (H and E staining; 200 μm region under 10× magnification of fluorescence microscope) after 24 h. The results were compared with ODL formulation and represented as: *P* < 0.05 (*), *P* < 0.01 (**), *P* <0.001 (***), *P* <0.0001 (****).

*In vivo* multispectral fluorescent imaging was used to evaluate DOX accumulation in internal organs and toxicity following i.v. administration into mice via the tail vein (Figure 9A). A high concentration of free DOX (red signal) was observed in all most all the major organs during the first quarter of the study period (4 h). A strong DOX signal (yellow/green) was observed in the mouse body after 24 h. These data indicated DOX was likely integrated with substrates in tissue and caused organ damage when administered at toxic doses (15 mg/kg, i.v.). Similar outcomes were observed with DXL. Unlike free DOX or DXL, a gradual distribution of DOX in the ODL was observed in the first half of the study period that increased and subsequently decreased. The lowest levels were observed in the heart and liver after 24 h (Figure 9A).

We evaluated oxidative stress in blood, plasma, and tissue (heart, liver and kidney). Reduced GSH-Px activity in blood and plasma was observed in mice treated with DOX (free DOX and DXL) compared to ODL (Figure 9B). Similarly, reduced GSH-Px activity was observed in liver homogenates from mice treated with DOX and DXL compared to ODL (p < 0.05) (Figure 9C). Reduced GSH-Px activity in the kidney was also observed in mice treated with DOX compared to ODL (Figure 9C). No differences in GSH-Px activity in the heart were observed in mice treated with DOX, DXL, and ODL (*p* > 0.05). The lowest GSH-Px activity was observed in DOX-treated mice (Figure 9C). GSH-Px activity in mice treated with OAL was similar to the controls.

GSH-Px activity in the heart was underestimated for ODL. There was no difference (*p* > 0.05) compared to the other treatments, with the exception of OAL. Therefore, we explored an indirect mechanism of heart injury through the arachidonic acid (AA) pathway. We measured the levels of 20-HETE, a metabolite of the AA pathway produced by CYP4A, in matrix samples (Figure 9D). Higher 20-HETE production was observed in all samples in DOX-treated mice. Similar results were observed for mice treated with DXL. Lower 20-HETE production was observed in mice treated with ODL. The lowest 20-HETE levels were observed in mice treated with OAL.

Histopathological changes in tissue samples were indicative of DOX-induced toxicity. We observed a reduction of striated muscle bands, congestion, rippled myocytes, myocytolysis, and hemorrhagic areas in heart tissue collected from DOX-treated mice (Figure 9E). Similar results were observed in mice treated with DXL. No visible tissue damage was observed in mice treated with OA, OAL, and ODL. Diminished Kupffer cells indicative of liver injury were observed in mice treated with DOX and DXL. No signs of liver injury were observed in the other groups. Hematoxylin and eosin (HE) staining revealed that control mice and mice treated with OA, OAL, or ODL had normal renal glomeruli and cortical tubule structures. However, glomeruli distortion, focal tubule atrophy and necrosis, the absence of filtration space, vascular congestion, and exfoliation were observed in the DOX-treated mice. The spleens of DOX-treated mice (DOX, DXL, and ODL) exhibited expanded splenic sinuses, swollen splenocytes, and congestion in the red pulp area. The extent of tissue injury was lower in mice treated with ODL. Mice treated with free OA or OAL had no signs of tissue damage. Histopathological changes were observed in the lungs of mice treated with DOX and DXL compared to controls. Mice treated with ODL, free OA, and OAL had normal lung tissue.

**Figure 10:**
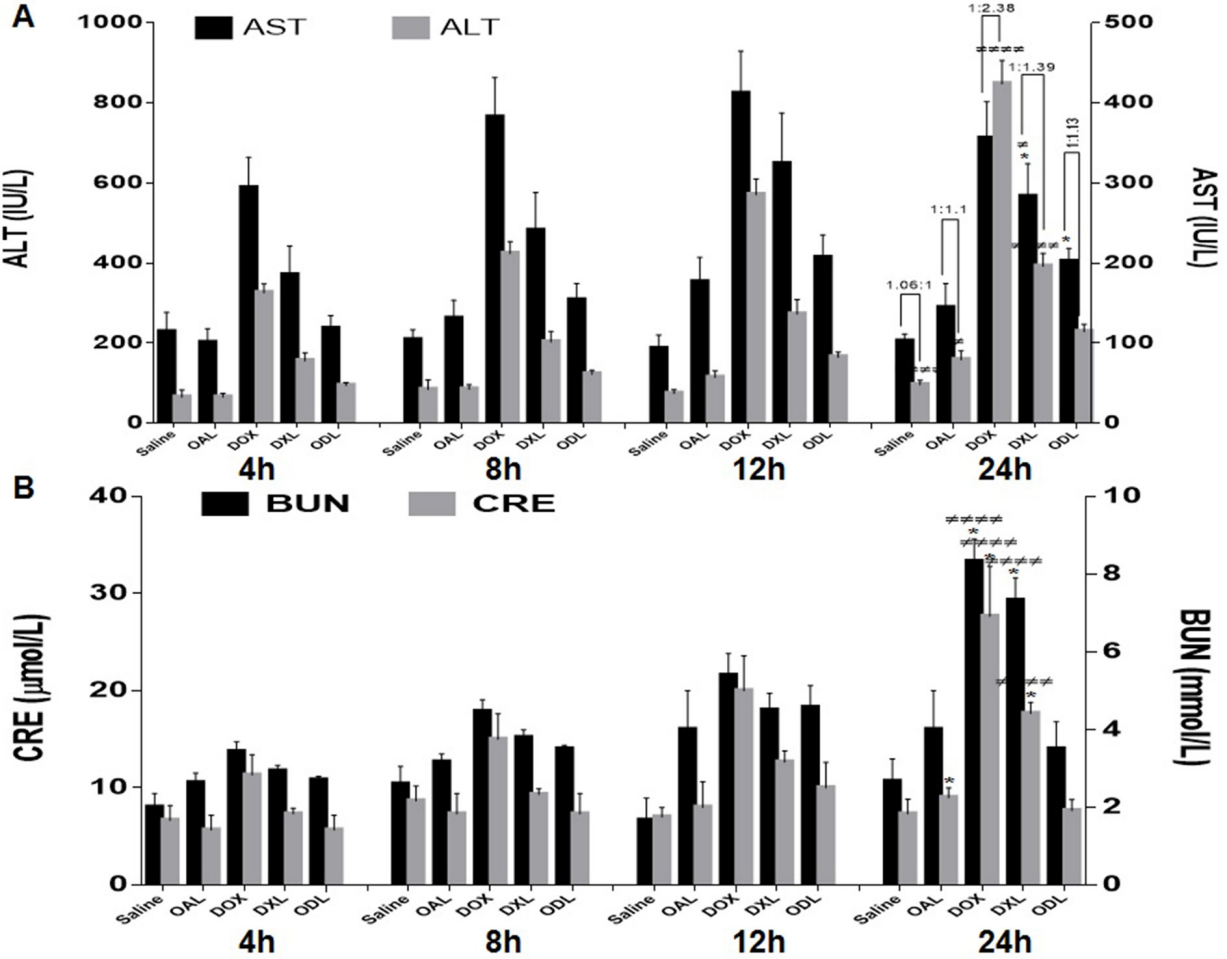
Time dependent DOX effect on biomarkers after i.v. administration of toxic dose (15 mg/kg) (*n* = 3) (**A**) Liver function test, AST:ALT ratio on horizontal bar (**B**) Kidney function test (BUN and CRE values).

Organ toxicity was also indicated by an increase in the levels of biochemical markers including aspartate aminotransferase (AST), alanine aminotransferase (ALT), blood urea nitrogen (BUN), and creatinine (CRE). A gradual increase in AST, ALT, BUN, and CRE levels was observed in DOX-treated mice indicating time-dependent tissue damage (Figure 10). AST levels suddenly decreased while ALT levels increased after 24 h, which was indicative of DOX-induced liver damage (1:2.38, AST:ALT, respectively) (Figure 10). The BUN and CRE levels were 3.43 ± 0.25 mmol/L and 7.33 ± 0.58 μmol/L, respectively, after 4 h of DOX exposure. The levels increased to 8.33 ± 0.58 mmol/L and 27.67 ± 5.13 μmol/L, respectively, after 24 h, which was indicative of kidney dysfunction. There was no difference in the levels of biochemical markers (with the exception of ALT) in mice treated with ODL and saline (control) after 24 h. Although ALT levels were higher after 24 h in mice treated with ODL compared to controls, there was reduced than DOX and DXL. No differences in BUN and CRE levels were observed after 24 h in mice treated with ODL compared to controls. These mice also had no changes in biomarkers compared to controls throughout the study period.

## DISCUSSION

OA has antiinflammatory, antiviral, hepatoprotective, antitumor, cardioprotective, and antihyperlipidemic effects. However, the clinical applications of OA are limited due to poor aqueous solubility. Nano-carrier systems have been developed to circumvent this issue [22–26]. We investigated co-delivery of OA and chemotherapeutics as a potential therapeutic strategy for HCC. OA and DOX had a synergistic effect in HepG2 cells. OA displayed free radical-scavenging activity with and without DOX (supplementary data). We hypothesized that OA could attenuate the cardiotoxicity of DOX. Multiple formulations of DOX and OA were analyzed using an orthogonal approach. The A2B1C1D2 model improved the EE of the loaded drugs, particle size limit, and controlled release of the drugs from liposomes.

We performed serum stability studies to understand the fate of the drug under physiological conditions. The amount of drug leakage in serum was quantified. Lipophilic drugs accumulate in the lipid compartment of liposomes and act as a barrier for hydrophilic drugs that accumulate in the inner aqueous core. The presence of OA in the outer lipid bilayer was advantageous given the sustained *in vivo* release of DOX. The circulation times of PEG-modified liposomes are independent of the lipid quantity and composition, surface charge, and PS [27]. The optimal size of PEG-conjugated liposomes for prolonged circulation is 160–275 nm [28]. Our liposomes were stable for 6 months. Drug release was evaluated in DMEM and under acidic or basic conditions to mimic the *in vivo* microenvironment. Controlled release of the drugs in all media analyzed demonstrated the stability of the formulations. The relatively fast DOX and OA release in DMEM could be explained by non-specific protein absorption and PEG aggregation, which perturb the integrity of the liposomes leading to drug release [29]. There was no initial burst in DOX release from DXL/ODL at pH 7.4 relative to free DOX, suggesting that the liposomes were stable under physiological conditions. An increase in drug release was observed at pH 4.5. Thus, DOX could be released under acidic conditions in endosomes and then enter the nucleus by diffusion. The release profile of OA was biphasic at physiological pH (7.4). A relatively large burst effect was observed followed by a slower release phase. The initial fast release rate corresponded to drug detachment from the outer surface, while slower release corresponded to drug release from the inner lamellae. OA was associated with the lipid bilayer. OA release followed a diffusion-controlled mechanism similar to its analog ursolic acid [30].

We observed an increase in the number of apoptotic bodies with combined delivery (i.e. ODL) compared to individual drug-loaded liposomes (i.e. DXL/OAL), indicating the drugs had synergistic anticancer effects. Induction of apoptosis was time-dependent. Increased apoptosis was observed as the exposure time was increased from 24 h to 48 h. LDH leakage is only observed upon the loss of cell membrane integrity. We performed LDH leakage assays to assess the cytotoxicity of the formulations in tumor cells *in vitro*. Less LDH release was observed during the first 24 h. The gradual increase in LDH leakage after 24 h was probably due to the initial interaction of the cell membrane with OA. Dissolution studies suggested a similar pattern of drug release from ODL (i.e. OA followed by DOX release at pH 7.4) (Figure 7B). These observations could also be explained by differences in the mechanisms of uptake of free drugs compared to liposomes.

The ODL formulation inhibited tumor growth and attenuated the toxicity of DOX. Combined delivery did not cause obvious toxicity to the liver, kidney, and heart, suggesting that OA had a protective effect. Therefore, treatment with ODL may be safer than treatment with either free DOX or DXL. The protective role of OA against DOX-induced toxicity was supported by the presence of both OA and DOX in all bio-compartments (Figure 7E). The ratios of the cumulative concentrations of both drugs in plasma, heart, liver, kidney, spleen, and lung were 1.49:1, 1.42:1, 0.8:1, 0.78:1, 0.57:1, and 0.47:1 OA:DOX, respectively. The higher concentration of OA in plasma and heart tissue suggested that OA protected against DOX-induced cardiotoxicity.

We investigated the cardioprotective effect of OA in H9C2 cardiomyocytes. OA reduces apoptosis in H9C2 cells at 100 μM [13]. The cytotoxic effect of DOX in H9C2 cells was demonstrate previously [31]. Cells were nearly 100% viable after treatment with 100 μM OA (Figure 8A). The CI values demonstrated antagonism between both drugs (Figure 8B). These data indicated that OA could attenuate DOX-induce cardiotoxicity.

We determined that OA stabilized antioxidant-oxidant equilibrium and restored DOX-induced suppression of GSH-Px activity. OA restored GSH-Px levels in blood, plasma, and vital organs through its free radical-scavenging ability. However, 20-HETE may have indirectly caused cardiotoxicity. AA is metabolized by CYP4A and 4F enzymes to generate 20-HETE in various organs such as the liver, kidney, heart, lung, brain, and vasculature. In the vasculature, 20-HETE is a potent vasoconstrictor. Upregulation of 20-HETE production results in increased ROS [32]. Higher levels of 20-HETE were observed in the heart, liver, and kidney after DOX exposure [11, 33].

We detected higher levels of 20-HETE in the heart, liver, and kidneys of DOX-treated mice, indicating DOX promoted organ damage by inducing 20-HETE production. A reduction in 20-HETE production was observed in vital organs of mice treated with OA and OAL compared to DOX (free and liposomal). A limited amount of 20-HETE metabolites were observed in mice treated with ODL. OA attenuated DOX-induced ROS and reduced the toxic effects of 20-HETE (ROS production and vasoconstriction in the myocardium) through free radical-scavenging. OA may also reduce other toxic effects of 20-HETE through overproduction of PGI_2_, a vasodilator prostanoid, or though metabolism of 20-HETE to generate less bioactive metabolites (e.g. 20-OH-PGE_2_ and 20-OH-PGF_2α_) [16].

**Figure 11:**
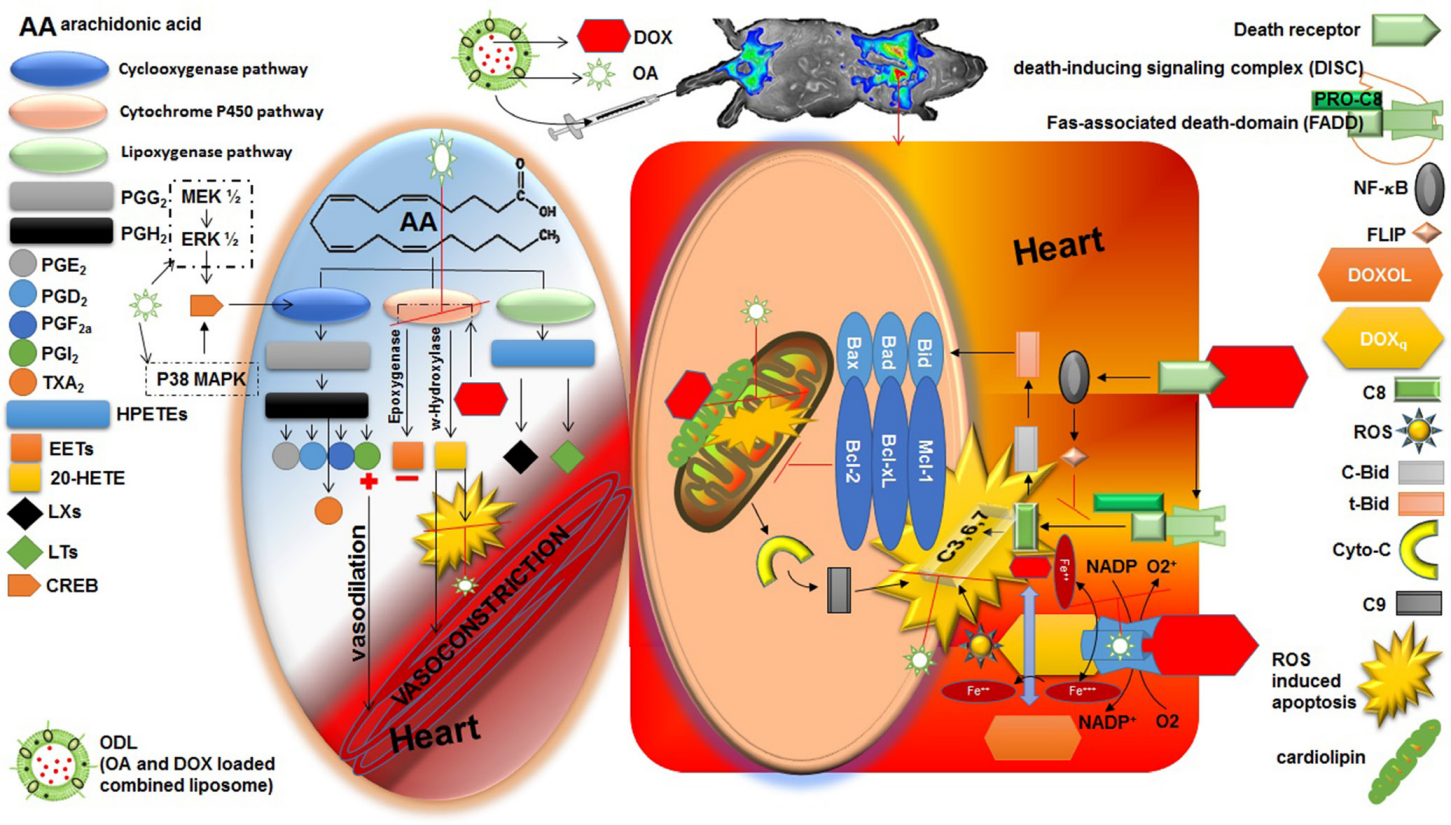
Cardio protective role of oleanolic acid (OA) against doxorubicin (DOX) induced cardiotoxicity Right side square box illustrated the direct route of DOX induced myocardiotoxicity. The left side oval shape illustrated the indirect route of DOX induced myocardiotoxicity. Heart is the most susceptible organ to attack by DOX. In the presence of NADH, the quinone moiety of DOX molecule is transformed into a semiquinone moiety. The semiquinone moiety reacts with molecular oxygen to form a superoxide radical (O^2-^), and DOX molecule returns to its original quinone form. This redox cycling generates a huge amount of superoxide radical (O2^-^) and thus in turn produces “supernova” of ROS. DOX also interferes with iron used for normal metabolic reactions and produces ROS. Caspase activity can also be influenced by DOX via several routes. Moreover, DOX has high affinity for cardiolipin, a phospholipid abundant in myocardium located on mitochondria, and disturbs mitochondrium function. Aside by its direct role in cardiotoxicity, DOX also affect the heart by formation of P450-derived arachidonic acid metabolites via significantly increased CYP4A expression. DOX act on expoxygenase enzymes to reduce their activity. Hence, epoxyeicosatrienoic acids (EETs) to 20-Hydroxyeicosatetraenoic acid (20-HETE) equilibrium is disturbed that lead to increase level of 20-HETE in the myocardium. 20-HETE injured the cardiomyocytes via several routes 1) ROS induction; 2) activating nuclear factor-κB (NF- κB); 3) increased caspase-3 activity. Aside by this, 20-HETE causes vasoconstriction in blood vessels. OA has potential to reduce the stress full condition (ROS burden) by its radical scavenging properties. OA has antioxidant and inhibitory effect on NF- κB that is advantageous to balance toxic consequences of DOX. Moreover, increased formation of PGE_2_ and PGI_2_ (vasodilator prostanoids) by activation of cyclooxygenase (COX) balanced the vasoconstriction response of 20-HETE. The time and dose dependent PGI_2_ production is associated with the upregulation of COX by OA.

DOX induces organ toxicity through multiple mechanisms. We combined DOX with OA, which reduced DOX-induced cardiotoxicity but did not inhibit the anticancer activity of DOX (Figure 11). The sustained release of the drug and the synergistic anticancer activity indicate ODL may be effective for HCC treatment. Reduced 20-HETE production in the heart compared to the liver, kidney, and plasma suggested the protective effect of OA was predominantly mediated by cytochrome P450 phospholipid metabolism in the heart. Additional studies are required to elucidate the mechanisms underlying OA-induced 20-HETE production and the roles of 20-HETE in carcinogenesis.

## MATERIALS AND METHODS

### Reagents

DOX-HCl was purchased from Beijing HuaFeng United Technology Co. (Beijing, China); OA and 2,2- diphenyl-1-picrylhydrazyl (DPPH) from Aladdin Industrial Corporation (Shanghai, China); dehydrogenated soya phosphatidyl choline (HSPC) from Shanghai Advanced Vehicle Technology Ltd. (Shanghai, China); cholesterol (CHOL) from Acros Organics (Morris Plains, NJ, USA); DSPE-PEG(2000) from Avanti Polar Lipids, Inc. (Alabaster, AL, USA); Sephadex G-25 from GE Healthcare Bio-Sciences AB (Uppsala, Sweden); Sepharose CL-2B from Beijing Solarbio Life Sciences, (Beijing, China); PBS and HEPES from Biosharp (Anhui, China); CHCl_3_, ethanol, methanol, (NH_4_)_2_SO_4_ (AR grade), and Tween 80 (CP grade) from Sinopharm Chemical Reagent Co. (Shanghai, China); acetonitrile and methanol (HPLC grade) from Thermo Fisher Scientific (Geel, Belgium); trifluoroacetic acid (TFA) from Merck (Darmstadt, Germany); MTT, DMSO, and DMEM from Sigma-Aldrich (St. Louis, MO, USA); FBS from Zhejiang Tianhang Biological Technology Co., Ltd. (Hangzhou, China); and DAPI from KeyGen Biotech. (Nanjing, China).

### Cell culture

The HepG2, HepG3B, H9C2, and L02 cell lines were obtained from the China Center for Type Culture Collection at Wuhan University (Wuhan, China). Cells were cultured in DMEM supplemented with 10% FBS (v/v), penicillin (100 units/mL), and streptomycin (100 mg/mL) in a 5% CO_2_ atmosphere at 37°C.

### Animal studies

Female Kunming mice were obtained from the Animal Care Facility of Huazhong University of Science and Technology (Wuhan, People's Republic of China). Six-week-old female BALB/c-nude mice (Beijing HFK Bioscience Co. Ltd, Beijing, China) were maintained in the animal care facility at Huazhong University of Science and Technology at 22°C ± 2°C. Mice were provided with water and food ad libitum. All animal protocols were approved by the Animal Experimentation Ethics Committee of Huazhong University of Science and Technology.

### Experimental design

DOX and OA doses were selected based on the doses that exhibited synergistic anticancer effects in HCC. OA was expected to reduce DOX-induced cardiotoxicity. We developed combined, nano-sized liposomal formulations of OA and DOX to gain the expected pharmacological effects. An orthogonal drug experimental design approach was used to optimize the formulations. Outcomes were investigated in a HepG2 mouse model of HCC. Toxicity was evaluated *ex vivo* and *in vivo*.

### Cell cytotoxicity and survival studies

### Analysis of cell viability and anticancer activity

Cell viability was analyzed after treatment of various cell lines (HepG2, Hep3B, and L02) with DOX (0.1–1 μg/mL) and OA (50–150 μg/mL). Cells were cultured at 37°C in a 5% CO_2_ atmosphere in 96-well plates at a density of 14,000 cells per well. The total volume per well was 100 μL. The media was replaced after 24 h with 200 μL of drug-containing media.

To evaluate the anticancer effects of the drugs, HepG2 cells were co-treated with DOX and OA at non-fixed and fixed ratios. For non-fixed ratio experiments, cells were treated with DOX at a concentration of 0.025 or 0.05 μg/mL in combination with OA at concentrations ranging from 50–150 μg/mL. Similarly, cells were treated with OA at a concentration of 25 or 50 μg/mL in combination with DOX at concentrations ranging from 0.1–1 μg/mL. For fixed ratio experiments, cells were treated with OA and DOX at a ratio of 2000:1 (i.e. 75:0.0375, 50:0.025, and 25:0.0125 μg/mL OA:DOX, [w/w], respectively).

Cells were incubated with the drugs for 72 h. Untreated cells reached > 80% confluence. Following the incubation, the cells washed twice with PBS and 200 μL of media containing 20 μL of MTT (5 mg/mL) added to each well. After incubating for an additional 4 h at 37°C in a 5% CO_2_ atmosphere, the media was replaced with 200 μL of DMSO to solubilize formazan crystals. The optical density at 490 nm was measured using a Multiskan MK3 microplate reader (Thermo Fisher Scientific, Atlanta, GA, USA). The median effect method [34] and CompuSyn software (version 1.0.1; CompuSyn Inc., Paramus, NJ, USA) were used to calculate the CI for drug combination studies. Interactions between OA and DOX were evaluated where synergy, additivity, and antagonism were defined as CI < 1, CI = 1, and CI > 1, respectively.

### Cell recovery assays

HepG2 cells were seeded in 24-well plates at a density of 14 × 10^4^ cells/mL and incubated at 37°C in a 5% CO_2_ atmosphere. After 24 h, the media was replaced with 500 μL of media containing DOX (0.1–1μg/mL), OA (50–150 μg/mL), or a combination of DOX and OA at a ratio of 2000:1 OA:DOX (w/w), respectively. After 72 h, the media was replaced with drug-free DMEM and the cells incubated for 72 h at 37°C in a 5% CO_2_ atmosphere. Following the incubation, 50 μL of MTT (5 mg/mL) was added to the each well and the cells incubated for 4 h. The media was replaced with 500 μL of DMSO to solubilize the formazan crystals and the optical density at 490 nm measured using a Multiskan MK3 microplate reader (Thermo Fisher Scientific, Atlanta, GA, USA).

### Liposome preparation

HSPC, CHOL, and DPSPE. PEG_2000_ were used at a molar ratio of 64:31:5, respectively. Liposomes were prepared using a reengineered ethanolic injection method. Lipids were dissolved in chloroform and the resulting solution evaporated under nitrogen gas. The thin film of lipids was redissolved in 2 mL of absolute ethanol (preheated to 48°C). This ethanolic lipid solution was injected into the middle of the 5 mL aqueous phase (10 mM HEPES) and the solution stirred continuously for 10 min. The dispersion was sonicated for 30 min using an Ultrasonic Cleaner (Ningbo Scientz Biotech. Co. Ltd, China). Homogenization and temperature affected drug loading and PS. Temperature was varied (43°C, 48°C, and 53°C) while the stirring time was held constant (45 min). Ethanol was removed by rotary evaporation under reduced pressure to generate the final dispersion, which was sonicated for 30 min to generate small multi-lamellar vesicles.

Lipid composition and ratio, drug-to-lipid ratio, ethanol concentration in the final formulation, and temperature were critical for appropriate drug loading and efficacy. Orthogonal experiments were designed at three levels to optimize the formulations (Table 1 and Table 2). All liposome dispersions were stored at 4°C. The OA was pre-dissolved in ethanol containing lipids to obtain OAL. DOX was loaded using an ammonium gradient at 60°C. A Sephadex G 25 column was used to establish a transmembrane sulfate gradient for the active loading of DOX. Dynamic light scattering (Nano Brook Zeta PALS, Brookhaven Instruments Corporation, Holtsville, NY, USA) was used to determine the mean diameter and PDI. The ZP was also measured.

### Drug loading and EE

Free OA was removed by slow speed centrifugation (Heraeus Multifuge X1R, Thermo Fisher Scientific, Darmstadt, Germany) at 3000 × g for 10 minutes. The supernatant containing OAL was collected in a separate vial. Free DOX was removed by passing the liposomes over a Sepharose CL-4B/CL-2B column. Liposomes were eluted in 10 mM HEPES buffer pH 7.4. Free DOX was retained in the gel. DXL migrated through the gel and were collected to generate the appropriate doses. The diluted liposomes were pooled and concentrated using Spectrum™ MicroKros Hollow Fiber Modules (Thermo Fisher Scientific, Rancho Dominguez, CA, USA).

OA was quantified by HPLC (Agilent 1220 Infinity LC System. Germany). The ChemStation software (Agilent) was used for data acquisition and analysis. The chromatography columns were the following: Agilent Zorbax SB-C18 (2.1 mm, 50 mm) and a Sepax Technologies Sapphire C18 analytical column (4.6 mm × 250 mm, 5 μm). OA was detected at a 210 nm at a flow rate of 1.3 mL/min. The mobile phase consisted of 0.1% TFA in water and a mixture of acetonitrile: methanol (17:1) at a ratio of 10:90. A standard curve of OA in methanol was generated for a range of concentrations (5–160 μg/mL). The DOX concentration in liposomes was measured using a Spectrum 756PC UV-VIS spectrophotometer (Shanghai Spectrum Instruments Co. Ltd., China) at 480 nm. A standard curve of DOX in methanol was generated for a range of concentrations (0.8–25.6 μg/mL).

The EE of the liposomes was calculated after lysis in methanol using the following equation:

%Drug loaded(%DL)=(amountofencapsulateddrug/amountoflipids)×100 1

%Entrapment Efficiency(%EE)=(amountofencapsulateddrug/amountofdrugfed)×100 2

### Liposome characterization

Liposome morphology was analyzed by TEM (Hitachi, Tokyo, Japan). A drop of each formulation was placed on a carbon-coated copper grid, which formed a thin liquid film. Excess solution was removed with filter paper and the samples air-dried prior to imaging.

To confirm the attachment of PEG to the liposomes, the lyophilized liposomes were studied by Fourier transform infrared spectrometry (supplementary file).

### Liposome stability studies

### Serum stability

Stability studies of DOX-loaded liposomes (DXL and ODL) in serum were performed as previously described [35]. The liposomal dispersion was incubated in 10 mM HEPES pH 7.4 with 20% FBS at 37°C in water bath (DF-101S; Yuhua Instrument Co., Ltd., Gongyi, China) to generate a final drug concentration of 100 μg/mL. The increase in florescence intensity due to release of free DOX from liposomes was measured at λ_excitation_ = 480 nm and λ_emission_ = 585 nm with a spectrofluorometer (F-2700, Hitachi, Tokyo, Japan). The percent drug release (% DR) was calculated using the following equation:

$$\%\text{DR}=\left(\left(F_{t-}F_{0}\right)/\left(F_{\max-}F_{0}\right)\right)\times 100$$ 3

F_0_, F_t_, and F_max_ denote the fluorescence intensities prior to the addition of liposomes (time 0), at specific time intervals (t), and the maximum after breaking the liposomes in methanol, respectively.

Serum stability studies of OA-loaded liposomes (OAL and ODL) was performed using a modified version of the protocol described by Li et al. [36]. Samples of concentrated, OALs were diluted in 20% FBS in 10 mM HEPES pH 7.4 to obtain a final drug concentration of 500 μg/mL. The samples were vortexed for 1 min and incubated at 37°C in a water bath. Separate samples were collected at various time intervals and centrifuged at 3000 × g for 10 min. Supernatants were collected and analyzed by HPLC. The % DR was calculated using the following equation:

$$\%DR=\left(\frac{A_{0}-A_{1}}{A_{max}}\right)\times 100$$ 4

A_0_, A_t_, and A_max_ denote the amount of drug at time 0, at time (t), and the maximum after lysis of the liposomes with methanol.

### Stability in storage

Liposome stability was evaluated at two different temperatures (4°C and 25°C) based on the remaining drug content, PS, and ZP. DOX was measured by UV and the remaining OA was assessed by HPLC.

### Drug release studies

Drug release was monitored in complete DMEM and 10 mM HEPES at pH 7.4 and pH 4.5. A dialysis bag (MWCO 3,500) containing 2 mL of the liposomal dispersions, equal to 1 mg/mL of DOX in DXL and 10 mg/mL of OA in OAL (concentrated liposomes), was immersed in 60 mL of dispersion media at 37°C ± 1 with mild stirring. A 2 mL volume of ODL was equal to 2 mg/mL DOX and 10 mg/mL of OA (OA:DOX, 5:1, w/w, respectively). A 500 μL sample was collected from the media at specific time intervals. The same volume was then replaced with fresh media. DOX release was analyzed using a spectrofluorometer while OA was quantified by HPLC. We added 1% Tween 80 in the dissolution medium to solubilize free OA in OA drug release studies.

### *In vitro* anticancer activity of liposomes

The cytotoxic effects of the liposomal formulations were evaluated using MTT assays. HepG2 cells were seeded in 96-well plates at a concentration of 14 10^4^ cells/mL in 100 μL of media. After 24 h, the media was aspirated and replaced with 200 μL of drug-loaded liposomes in media [DXL (0.05–20 μg/mL), OAL (50–1250 μg/mL), and ODL (5:1 OA:DOX, w/w)]. The cells were then incubated for 72 h at 37°C in 5% CO_2_ atmosphere. Next, 20 μL of MTT solution (5 mg/mL) was added to each well in the dark and the cells incubated for 4 h at 37°C in 5% CO_2_ atmosphere. The media was removed and 200 μL of DMSO added to dissolve formazan crystals. The plates were read on an ELISA reader at 490 nm and the percent cell viability measured.

### Apoptosis assays

A total of 14 10^4^ cells/mL were seeded in 24-well plates (500 μL per well). After 24 h, the cells were exposed to various drugs at the IC_50_. Saline was used as a control. After 24 h or 48 h, the cells were washed with PBS (1 mL) and harvested in binding buffer (400 μL) containing 5 μL of annexin V-FITC and 10 μL of PI. Cells were then incubated for 15 min at 37 °C.

After treatment with DXL and ODL for 24 h or 48 h (the exposure times for free DOX were 4 h and 12 h), the cells were stained with DAPI for 5 min and then washed with PBS. The cells were then resuspended in 400 μL binding buffer containing 5 μL of annexin V-FITC and incubated for 15 min at 37°C. Stained cells were analyzed by flow cytometry using a FACSCalibur instrument (BD Biosciences, San Jose, CA, USA). Quadrants contained the following cells: upper left quadrant (Q1), primary necrotic cells; upper right quadrant (Q2), late apoptotic cells; lower left quadrant (Q3), living cells; lower right quadrant (Q4), early apoptotic cells.

### *In vivo* antitumor assays and end-point bio-distribution studies

The i.v. route was disadvantageous because of the low solubility of OA and the robust *in vitro* release of OA at physiological pH (7.4). However, the majority of DOX administered i.p. remained in the abdominal cavity [37]. The accumulation of DOX in tissue after repeated injection can cause tissue damage. We utilized a modified i.v. followed by i.p. protocol for combined delivery of ODL [38, 39]. This protocol reduced the accumulation of the drug at the injection site, which could cause venous blockage as a result of insoluble OA or tissue damage caused by DOX deposition.

We subcutaneously injected 100 μL of HepG2 cell suspension (4 × 10^7^) into the backs of female BALB/c nude mice (approximately 20 g, 6–8 weeks old). Once tumors were visible, the mice were randomly distributed into six groups (three mice per group) and were i.v. injected with DOX (7 mg/kg) and OA (35 mg/kg), either in free or liposomal form, via the tail vein. Four doses were administered every other day. Three additional doses were i.p. injected every fourth day. The dose in ODL was 20:4 mg/kg OA:DOX, respectively, which resulted in synergistic antitumor effects. Tumor length and width were measured on alternate days using vernier calipers and the body weight of the mice recorded. Tumor volume (V) was calculated using the equation: V = (width^2^ × length)/2. Approximately 500 μL of blood was collected via the eyeball in heparin-treated tubes 24 h after the last injection. The samples were centrifuged at 5,000 rpm for 5 min to collect the plasma. The mice were then sacrificed and tumors dissected. Tumor weight was measured and the tumors photographed. Vital organs (e.g. heart, liver, kidney, lung, and spleen) were also collected. Drug levels were quantified in the blood and tissue samples. The ratios of liver, kidney, and heart weight to body weight (the organ index) were calculated using the following equation:

$$Organindex=\frac{wtoforgan\left(mg\right)}{bodywt.\left(g\right)}$$ 5

For drug quantification, 100 μL plasma samples in 1.5 mL Eppendorf tubes and 400 μL of extraction buffer (0.3 M HCl:Ethanol, 3:7, v/v) added. The solutions were vortexed for 5 min and then centrifuged at 12,000 rpm for 5 min. Supernatants were collected and stored at 4°C. We weighed 100 mg tissue samples and then minced the tissue in the extraction buffer (4 mL). Samples were then centrifuged at 12,000 rpm for 5 min and the supernatants collected. Supernatants were stored at 4°C prior to analysis. The DOX concentration was estimated by fluorescence spectrophotometry at 485/585 nm. Extracted samples were pooled for OA analysis and evaporated under a rotary evaporator followed by nitrogen flux at 60°C. Samples were stored at -20°C until analysis. Resin was resuspended in methanol (100 μL, HPLC grade) and centrifuged at 1,2000 × g for 10 min prior to HPLC. Supernatants were collected and analyzed by HPLC to determine the OA concentration.

### Toxicity evaluation

### *Ex vivo* toxicity

The IC_50_ of each formulation in H9C2 cells was evaluated using MTT assays. H9C2 cells were cultured in 96-well plates at a density of 14,000 cells per well and allowed to adhere for 24 h at 37°C in a 5% CO_2_ atmosphere. The cells were treated with free DOX or OA, FDC, and ODL. The drug ratios in the FDC and ODL were 200:10 μM, 200:20, and 200:40 OA:DOX, respectively), which corresponded to non-fixed ratios of 20:1, 10:1, and 5:1, respectively. After 48 h, the media was aspirated and replaced with MTT-containing media (5 mg/mL), and the cells incubated for an additional 4 h at 37°C. Following the incubation, the media was removed and the formazan crystals dissolved in 200 μL of DMSO. The optical density at 490 nm was measured with a microplate reader (Multiskan MK3; Thermo Fisher Scientific, Atlanta, GA, USA). The dual-effect of the drugs was estimated in term of the CI values, which were calculated in HepG2 cells.

A commercial LDH kit (Jiancheng, Nanjing, China) was used to determine amount of LDH leakage in the extracellular medium of HepG2 cells. After drug treatment (dose = IC_50_) for 24, 48, or 72 h, the media was collected and LDH release assessed by measuring the absorbance at 440 nm using a Spectrum 756PC UV-visible spectrophotometer (Wincom Company Ltd., Hunan, China), according to the manufacturer's protocol. The levels of GSH-Px in the media were measured after drug treatment for 24 h (dose = IC_50_) using the manufacturer's protocol (Nanjing Jiancheng Bioengineering Institute, Nanjing, China).

### *In vivo* toxicity

DOX localization was analyzed in various bio-compartments at a toxic dose (15 mg/kg). Female Kunming mice (approximately 20 g, 6–8 weeks old) were divided into five groups: I- saline control, II- OAL (75 mg/kg), III- free DOX (15 mg/kg), IV- DXL (15 mg/kg), and V- ODL (OA = 75 mg/kg, DOX = 15 mg/kg). The appropriate dose was i.v. injected through the tail vein. At specific time intervals (4 h, 8 h, 12 h, and 24 h), the mice were anaesthetized for 10–20 min with 2.5% isoflurane in O_2_ and imaged by fluorescence using an In-Vivo FX Pro imaging system (Bruker Corp. Billerica, MA, USA) at 485/600 nm. Approximately 1 mL of the blood was collected from the orbital sinus after recovery from the anesthesia. Samples were centrifuged at 5,000 × g for 5 min. ALT, AST, BUN, and CR levels in plasma were analyzed using a Roche ISE900 automatic biochemical analyzer (Roche, Basel, Switzerland). Finally, the mice were sacrificed and vital organs weighed, fixed with 10% formalin, and stained with HE for pathological analysis.

An additional set of female Kunming mice was divided into five groups and similar doses administered. After 24 h, GSH-Px activity was assessed in whole blood, plasma, and tissue (e.g. heart, liver and kidney). We quantified 20-HETE in plasma and tissue samples using ELISA assays according to the manufacturer's protocol (Shanghai Jiang Lai Biology Technology Co. Ltd., Shanghai, China).

### Statistical analysis

Data are presented as the mean ± standard deviation (SD). Unless otherwise stated, statistical evaluations were performed using analysis of variance followed by Dunnett's multiple comparison test and GraphPad Prism Version 6.00 (San Diego, CA, USA). A *p* < 0.05 was considered statistically significant.

## SUPPLEMENTARY MATERIALS FIGURES


