# Supplementary material for: Liposomal co-delivered oleanolic acid attenuates doxorubicin-induced multi-organ toxicity in hepatocellular carcinoma
Source: Oncotarget. 2017 May 2;8(29):47136–53. doi: 10.18632/oncotarget.17559 (PMC5564550; doi:10.18632/oncotarget.17559)
Supplement: Supplementary file 1 [file oncotarget-08-47136-s001.pdf]

Liposomal co-delivered oleanolic acid attenuates doxorubicin-induced multi-organ toxicity in hepatocellular carcinoma

Supplementary Materials

Radical scavenger activity

The scavenging activities of OA were investigated in-combination with DOX. An ethanolic solution of drug sample (1 mL) was mixed with freshly prepared 0.1 mM DPPH radical ethanolic solution (1 mL). The mixture was vortexed for 30 seconds and kept in the dark for 30 min to complete the reaction. The absorption was recorded at 517 nm using UV-Vis spectrophotometer. Ascorbic acid was used as a positive control. The radical scavenger effect of OA was manifested by reduction in the absorption. The percent of scavenging was measured as % of DPPH radical activity by the equation:

$$\%\,DDPH\ Activity = \frac{Absorption(control) - Absorption(sample)}{Absorption(control)} \times 100 \qquad 1$$

Results of radical scavenging activities showed that the OA has moderate radical scavenging potential with 1 mg of OA resulted 19.21 ± 3.45% of ROS scavenging, which was equal to 2.55 ± 0.26 µg/mL of ascorbic acid used as positive control. No significant change was observed on the scavenging activity of OA in the presence of DOX at the weight ratio of 5:1 (OA:DOX, respectively) ($p > 0.5$) as shown in Supplementary Figure 1.

Fourier transform infrared spectrum analysis

A small quantity of lyophilized liposomes was amalgamated with dry potassium bromide (Kbr). The mixture was then ground into fine powder with the help of mortar and pestle. The fine pellet of this powder sample was prepared under hydraulic press at 10,000 psi. Infrared spectra of empty liposome was recorded by fourier transform infrared spectroscopy (FTIR) (Bruker VERTEX 70 FTIR spectrophotometer) at the range of 4000 cm^{-1} to 400 cm^{-1}. The same procedure was repeated with pure DSPE.PEG$_{2000}$ sample.

The presence of the DSPE.PEG was manifested by the characteristic carbonyl keto band at 1739 cm^{-1}. A CH alkyl stretching band was also observed at 2918/2919 cm^{-1} and at 2850 cm^{-1}. Broad strong bands were seen between 3400 cm^{-1} to 3450 cm^{-1} which corresponds to stretching vibration of the intramolecular hydrogen bond ($\upsilon_{O\text{-}H}$) of PEG. Moreover, a typical absorption band at approximately 1111 cm^{-1} was observed that was associated with C–O–C stretching vibration of the repeated –OCH_2CH_2– units of the PEG backbone. The FTIR of pure PEG (Supplementary Figure 2A) showed a series of bands that were also evident in the PEG coated liposomes (Supplementary Figure 2B) i.e. bands at 1739, 1636, 1542, 1148, 1111, 1063, 971, 722 and 529 cm^{-1}.

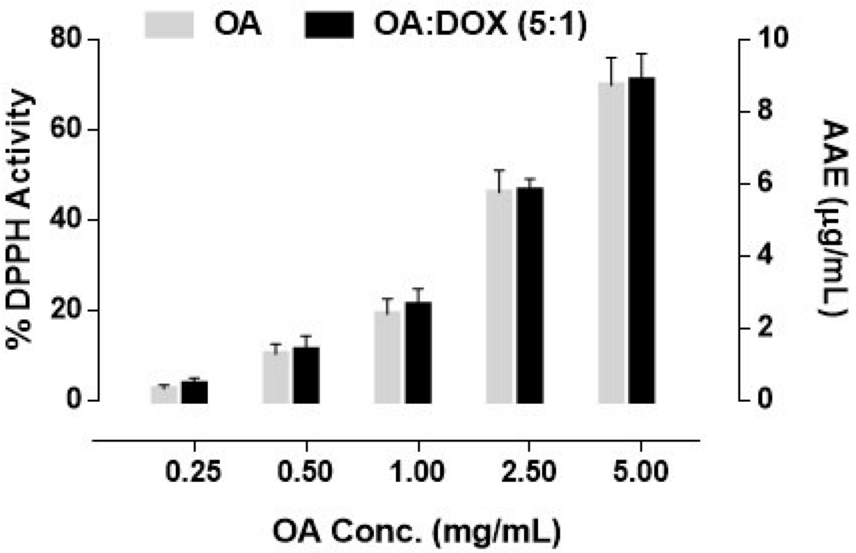

Supplementary Figure 1: Dose-dependent scavenging potential of OA with and without DOX in term of % DPPH activity with AAE as standard (*n* = 3).

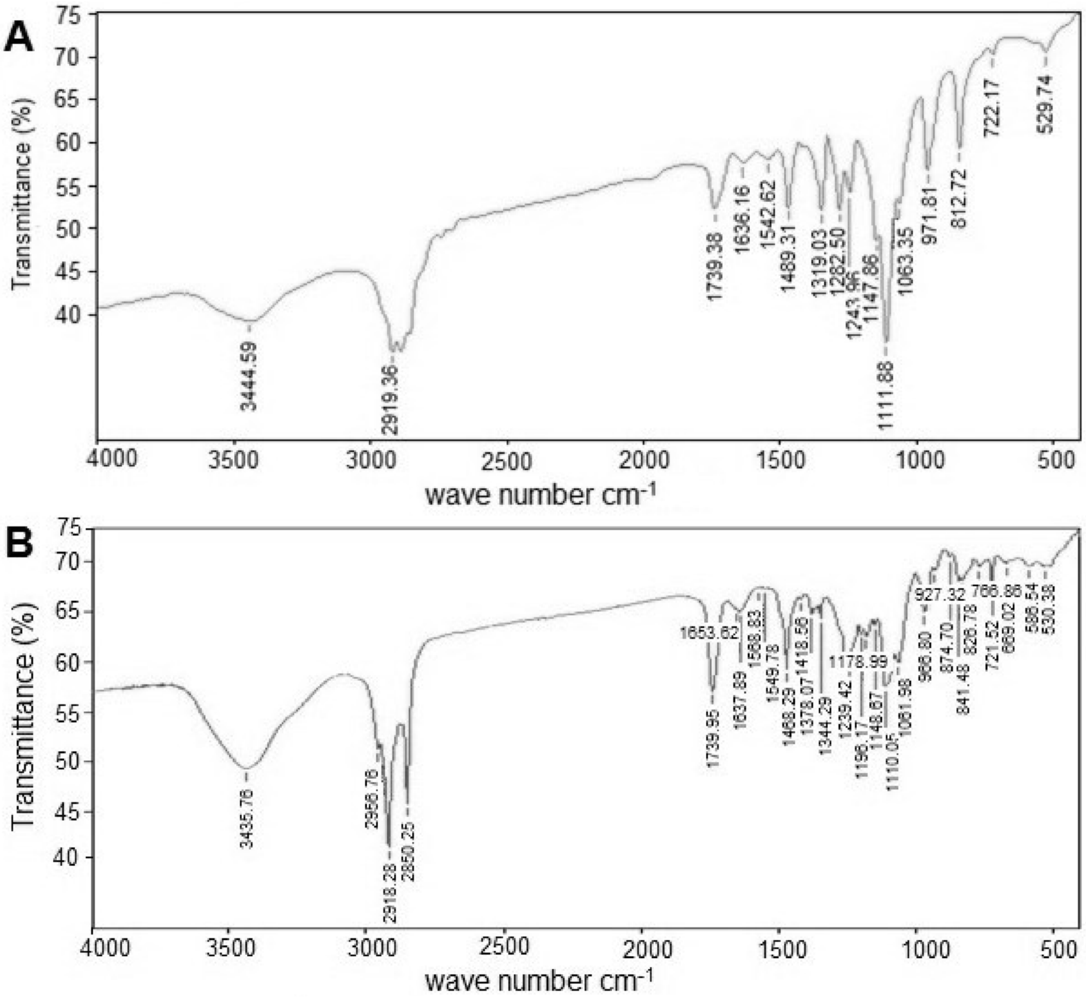

Supplementary Figure 2: Fourier transform infrared spectra of (**A**) pure DPSE.PEG$_{2000}$ powder (**B**) Lyophilized liposomes.
